# NUTMEG: Open Source Software for M/EEG Source Reconstruction

**DOI:** 10.3389/fnins.2020.00710

**Published:** 2020-08-25

**Authors:** Leighton B. N. Hinkley, Corby L. Dale, Chang Cai, Johanna Zumer, Sarang Dalal, Anne Findlay, Kensuke Sekihara, Srikantan S. Nagarajan

**Affiliations:** ^1^Department of Radiology and Biomedical Imaging, University of California, San Francisco, San Francisco, CA, United States; ^2^Department of Psychology, University of Birmingham, Birmingham, United Kingdom; ^3^Aarhus University, Aarhus, Denmark; ^4^Tokyo Medical and Dental University, Tokyo, Japan

**Keywords:** software, source space analysis, MATLAB, electroencephalography, magnetoencephalography

## Abstract

Neurodynamic Utility Toolbox for Magnetoencephalo- and Electroencephalography (NUTMEG) is an open-source MATLAB-based toolbox for the analysis and reconstruction of magnetoencephalography/electroencephalography data in source space. NUTMEG includes a variety of options for the user in data import, preprocessing, source reconstruction, and functional connectivity. A group analysis toolbox allows the user to run a variety of inferential statistics on their data in an easy-to-use GUI-driven format. Importantly, NUTMEG features an interactive five-dimensional data visualization platform. A key feature of NUTMEG is the availability of a large menu of interference cancelation and source reconstruction algorithms. Each NUTMEG operation acts as a stand-alone MATLAB function, allowing the package to be easily adaptable and scripted for the more advanced user for interoperability with other software toolboxes. Therefore, NUTMEG enables a wide range of users access to a complete “sensor-to- source-statistics” analysis pipeline.

## Introduction

Over the past several decades, magnetoencephalography (MEG) has emerged as an efficient technique to study brain function non-invasively with a high temporal resolution. As a result of this utility, a series of software packages have emerged over the same period of time that allow users to analyze this data [most notably FieldTrip (Oostenveld et al., 2011), minimum-norm estimation (MNE; Gramfort et al., 2013), and Brainstorm (Tadel et al., 2011)] and extensions of existing functional neuroimaging toolboxes in order to include MEG analyzes (such as SPM) have been developed. While popular, many of these toolboxes and approaches adapt existing techniques used for the analysis of electroencephalography (EEG) sensor data and apply them to the analysis of MEG data. While this approach is theoretically sound, it can be limiting, often restricting the user to techniques like sensor averaging, dipole fitting, magnetic field topography, and frequency decomposition at the sensor timeseries level.

More recently, adaptive spatial filtering (e.g., “beamforming”) analytic techniques have been developed in order to capitalize on the exquisite temporal resolution of MEG by providing an ability to localize where changes in MEG sensor data originate along the cortical mantle. These “source space” reconstruction techniques include a variety of spatial scanning estimates (such as minimum-variance adaptive beamforming (MVAB; Sekihara and Nagarajan, 2008), and synthetic aperture magnetometry (SAM; Vrba and Robinson, 2001), and tomographic reconstruction techniques including MNE (Hamalainen and Ilmoniemi, 1984), and standardized low-resolution brain electromagnetic tomography (sLORETA; Pascual-Marqui, 2002). While each of these inverse solutions are validated, robust techniques for source estimation, many data analysis packages implement only a few of these modeling techniques, and do not embed the ability to compare and contrast between different techniques.

Our goal is to provide a data analysis “workbench” that allows the user to compare and contrast different source modeling methods that may be the most appropriate for their dataset. In order to meet this demand for a flexible, easy-to-use, inverse-method inclusive MEG data analysis package we developed the Neurodynamic Utility Toolbox for Magnetoencephalo- and Electroencephalography (NUTMEG; Dalal et al., 2004) at the UC San Francisco Biomagnetic Imaging Lab. Originally released in 2003 and now on its fourth version, NUTMEG is an open-source, freely available MATLAB (Mathworks, Natick, MA, United States) based toolbox designed for M/EEG data analysis distributed for non-commercial use under a BSD-style license (The Open Source Initiative, 2004). It stands as a start-to-finish (or, “sensor-to-statistics”) pipeline of data analysis, capable of importing raw sensor data to running group-level statistics and functional connectivity (FC) analyzes. Each function in NUTMEG is a stand-alone command line function (akin to other software packages such as FieldTrip) allowing for easy batch scripting and custom pipeline development. In complement to that, these functions are assembled in a series of easy-to-use GUI interfaces that allow a more introductory user (e.g., technicians, clinicians, and students) to analyze full MEG studies with little necessary knowledge of command-line scripting. Visualization based on the SPM engine allows for ready navigation of source-space reconstructions. As the mission of NUTMEG development is to provide a variety of inverse method solutions to users across all levels of experience, it remains unique as a workbench given its vast array of source imaging methods available to the end user. The NUTMEG workbench ranges from gold-standard methods of source reconstruction (e.g., MNE, sLORETA, and beamforming) to more novel inverse methods developed in our lab [e.g., Champage, SAKETINI, and Covariance Optimization Garnering Noise for Active Cancelation (COGNAC)]. This separates NUTMEG from other workbenches given its unique ability to readily switch between different methods for all users to choose which inverse method is appropriate for their own unique studies. Our philosophy is to build a complete, stand-alone data analysis package for M/EEG data that appeals to a wide range of users with little dependency on outside software for running a scientific study.

Since the original version of NUTMEG and publications promoting its release, there have been substantial changes to the analysis workbench that provide more options to the user along the lines of source reconstruction, statistics, and FC analyzes. Our goal in the following article is two-fold. First, we outline some of the existing and additional, newer features of the workbench. Our focus is to provide new users an overview of how the NUTMEG process operates, from the pipeline itself to the MATLAB machinery “under the hood,” in a framework that allows both new and experienced users of M/EEG data analysis to utilize in a straightforward manner. Second, we present NUTMEG in a “how-to” format explaining how the standard NUTMEG data analysis pipeline is executed, from data import to analysis, using specific examples. We will go over the wide variety of options available in NUTMEG for both the introductory and advanced user, from the types of preprocessing steps available to inverse method solutions to choices of statistical tests.

## Getting Started

Neurodynamic Utility Toolbox for Magnetoencephalo- and Electroencephalography is available for download at the NeuroImaging Tools and Resources Collaboratory (NITRC) website^1^. NUTMEG is primarily written in MATLAB and has few dependencies on secondary software packages. NUTMEG has been tested on and currently operates efficiently in the most recent versions of MATLAB (at the time of this article: R2018a) although previous versions of NUTMEG are available for download at the NITRC website for compatibility with previous MATLAB versions. For digital filtering operations, the MATLAB Signal Processing Toolbox is required, and the Image Processing Toolbox is optionally needed for volume-of-interest (VOI) definition (described in Section Step 2. NUTMEG uses the SPM8 engine for visualization purposes^2^. Import of data formats from other software packages (such as FieldTrip) require installation of that software in the user’s MATLAB environment. Optional, third-party toolboxes are also available across the web by other developers that allow for automated artifact detection and rejection, boundary element modeling approaches, and others (Dalal et al., 2011).

## Workflow

**FIGURE 1:**
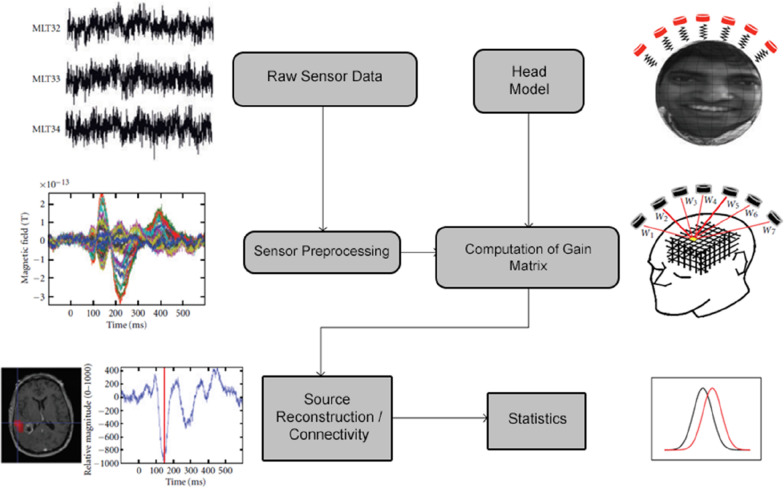
Outline of the standard NUTMEG data analysis pipeline for a single subject. Figure adapted from Dalal et al. (2011).

A schematic of the typical data analysis pipeline implemented in NUTMEG is shown in Figure 1. Briefly, raw sensor data (or sensor data pre-processed elsewhere) is read into the MATLAB environment and constructed into the NUTMEG variable structure. Following data import, a series of preprocessing steps (including channel selection and bandpass filtering) are available in NUTMEG for the user to prepare the raw data for source analysis. In parallel, a subject-specific head model is imported from the proprietary software associated with the MEG acquisition for lead field/gain matrix generation (spatial filter weights) necessary for source reconstruction, and an anatomical MRI brain template (generally a T1-weighted anatomical MRI specific to the subject) is imported for visualization purposes.

Once the MEG data and associated structural elements are imported, a variety of time-series analysis, time-frequency analysis, and FC source estimation options are available for the user (see Table 1). Following source space estimation and reconstruction, their result is saved out as a separate file for visualization on the MRI template. In the case of studies with large (*n* > 5) samples, a statistical workbench within NUTMEG is available for both looking at within-group/session effects as well as comparisons between pre-defined independent variables using voxelwise statistics. Options for visualizing both individual and group results on either canonical MNI templates or brain renderings are available within NUTMEG, as well as options for the user to export the data in different formats (Cartool, ANALYZE) for the purposes of overlay and rendering in the user’s visualization tool of choice (e.g., mri3dX, MRICro, and BrainnetViewer).

**TABLE 1:** List of denoising and inverse modeling methods available in NUTMEG.

**(A) Denoising options**
Stimulus evoked factor analysis (SEFA)
SEFA ICA
DSSP
Variational bayesian factor analysis (VBFA)
**(B) Inverse modeling methods — time-series**
BF Lf error vector
Beamspace/Beamspace noES
Champagne
Smooth champagne
COGNAC
Correlate columns
Eigenspace scalar/Vector beamformer
LCMV Scalar/Vector beamforner
MinNorm/MinNorm scalar
NSEFALoc
Point/Region suppression
Saketini
Thresholded lead field
AGMN-RUG
dSPM
sLORETA
swLORETA
**(C) Inverse modeling methods — time-frequency**
LCMV Scalar/Vector beamforner
MinNorm/MinNorm scalar
sLORETA
dSPM
SAM

## Gui Environment

**FIGURE 2:**
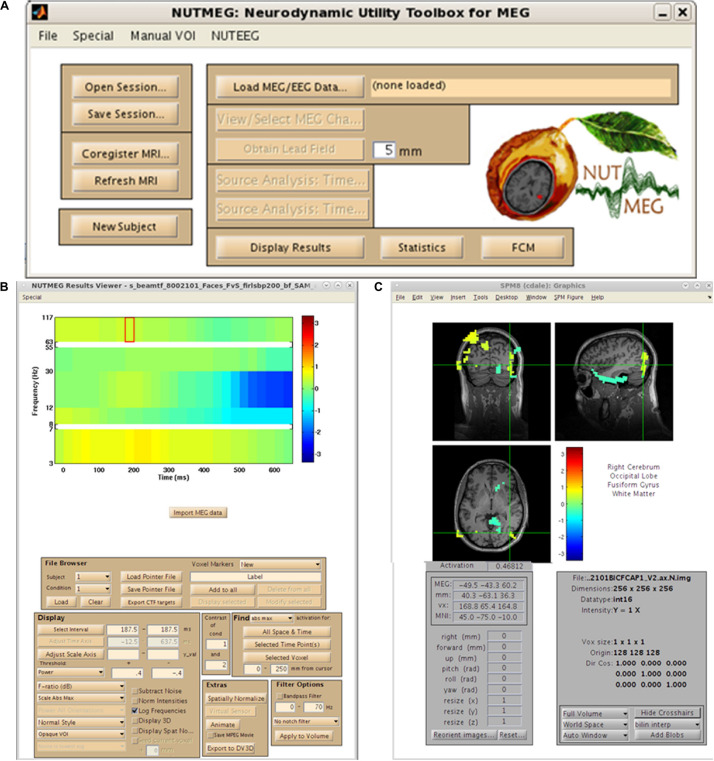
NUTMEG graphical user interface (GUI). **(A)** NUTMEG Main Command Window, **(B)** NUTMEG Results Viewer. **(C)** SPM Visualization Engine.

As mentioned earlier, the organization of NUTMEG as a series of stand-alone command-line functions not only make it ideal for the custom user who desires batch scripting and construction of analysis pipelines, but allows it to be easily built into a GUI-based environment where each function can be selected in point-and-click format. An example of this environment is shown in Figure 2. Here, we can see that all of the options laid out in the workflow (Figure 1) are selectable. The main NUTMEG GUI interface consists of three primary windows: the Main Command Window (Figure 2A), the NUTMEG Results Viewer (Figure 2B), and the SPM8 Visualization Engine (Figure 2C). From this point forward, we will present the series of steps necessary for the GUI-based user to execute a standard NUTMEG analysis.

## Data Import

Neurodynamic Utility Toolbox for Magnetoencephalo- and Electroencephalography has the capability to import raw data types from a variety of several major MEG manufacturers. This is demonstrated in the menu generated from the first analysis step, selection of the “Load MEG/EEG Data” button in the Main Command Window (Figure 2A). Here, we see options for CTF, 4D/BTi, KIT/Yokogawa/RICOH, and Elekta Neuromag data formats. These options will call one of the specific functions that are in standard use for MEG data import of these types (such as the ctf_read.m function for CTF datasets). Datasets can be either single- or multi-trial, and either raw or pre-processed (such as epoched and artifact-rejected) data in any of these formats can be imported into the toolbox. Datasets which have been cleaned in other software packages prior to data import (e.g., CTF DataEditor, ICA in EEGLab) are importable as well. In addition, several other options are available for import, including reading in data from FieldTrip file structure from EEG/MEG datasets. This converts the FieldTrip *fileio* data structure (Oostenveld et al., 2011) into the variable structure necessary for MATLAB to perform analyzes.

## Anatomical MRI and Fiducial Import, VOI Definition

**FIGURE 3:**
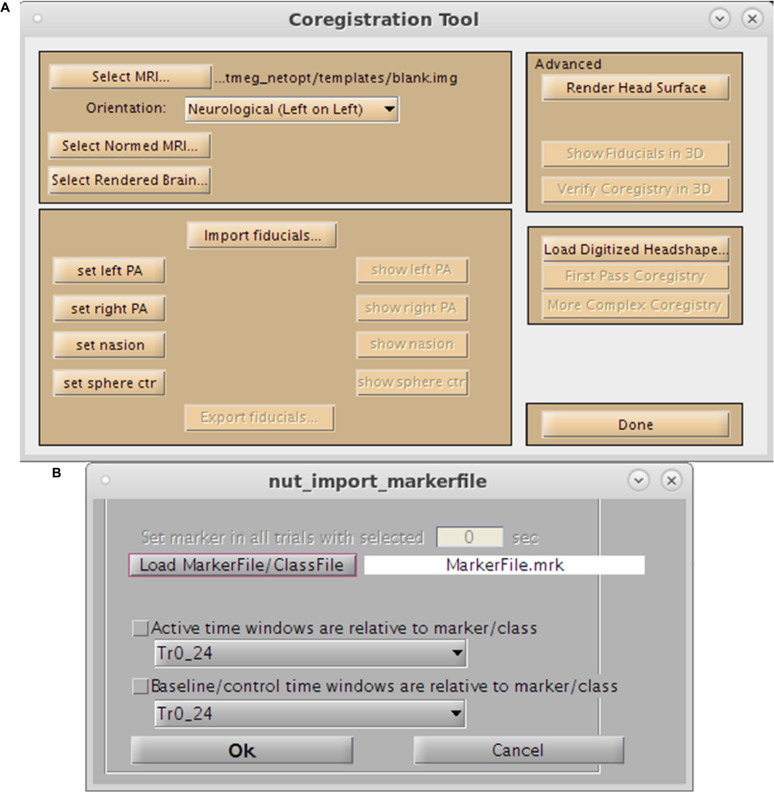
Preprocessing Toolbox GUIs. **(A)** Coregistration Toolbox GUI. Options include loading of the single-subject MRI, normalized MRI, fiducial assignment, importation of surface mesh, or headshape points. **(B)** Marker selection tool.

For purposes of source space imaging, the user is required to import a structural MRI and its associated head model information using the “Coregister MRI…” button on the Main Command Window (Figure 2A). This brings up the Coregistration GUI in a separate window (Figure 3). Through SPM8, NUTMEG is able to load both ANALYZE (^∗^.hdr/^∗^.img) and NIFTI (^∗^.nii) file formats for coregistration. The option of loading an additional, spatially normalized MRI associated with the individual allows for the computation of MNI coordinates for inter-subject comparison later in the analysis pipeline. To align the M/EEG sensor array with the structural MRI image, fiducials can be manually marked on the imported MRI within either the acquisition software (e.g., MRIViewer in CTF, MRILab in Neuromag), or NUTMEG itself, then co-registered with MEG coil positions imported from saved text files or the head model file created using menu options within the proprietary MEG acquisition software (i.e., MRIViewer, MRILab) or command line functions, such as localSpheres in CTF.

By default, the inverse solutions implemented in NUTMEG localize source activity across the whole brain volume, defined through a back transformation of labeled points in brain space in the spatially normalized structural MRI. However, it is also possible for the user to define an *a priori* VOI restricting source localization to a specific region of the anatomical volume. This is done from the “Manual VOI” drop-down in the main NUTMEG GUI, which allows the user to manually draw a three-dimensional polygon across the three anatomical orientations in the MRI. This VOI could include a specific sub-region of the brain (e.g., perilesional tumor tissue), a single hemisphere or restrict source localization to brain regions exclusively. One practical application of this is through the coherent source suppression approach detailed in Dalal et al. (2006), where in the case of highly temporally correlated sources (primary auditory cortex, localized through the M100 auditory evoked field) suppression of a single hemisphere permits more accurate source localization in the hemisphere of interest. VOIs defined through these custom definitions can then be saved out in the GUI for future reference.

## Pre-Processing and Channel Selection

**FIGURE 4:**
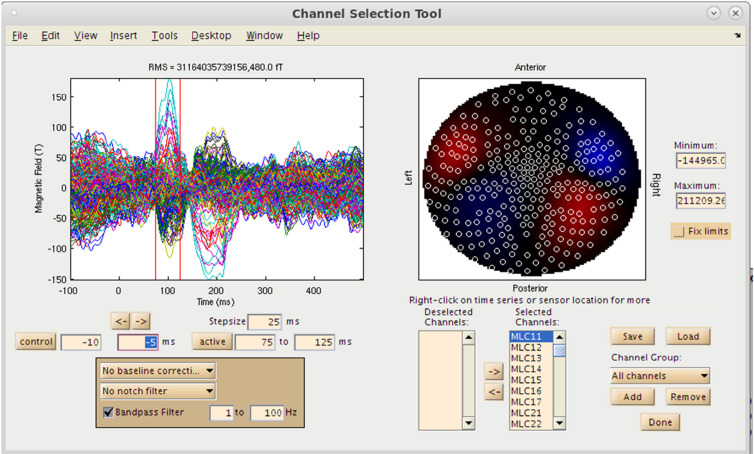
Sensor Preprocessing GUI. On the left, sensor overlay of the averaged dataset is visible. Over the right, RMS distribution over a selected time window is displayed. Options for channel selection, filtering, and denoising are available as well.

With both the MRI and MEG data loaded into the MATLAB environment, the user now has the option to visualize the MEG sensor data by selecting the “View/Select MEG Channels…” button on the Main Command Window, which brings up the Channel Selection interface in a separate window (Figure 4). Several options are available at this pre-processing step for the user to assess data quality prior to source imaging. Options for channel selection (inclusion/exclusion of channels in the analysis) are available in the dataset, and various filtering options (bandpass/notch filtering). Following selection of a time window of interest, the root mean square (RMS) value of the sensors selected will be displayed on a 2D sensor map.

Regularization and denoising techniques are available to remove artifacts prior to source localization in time-series/frequency analyzes. These include both approaches based off of ICA (SEFA-based ICA). Bayesian factor analysis (Nagarajan et al., 2007) algorithms are implemented in NUTMEG, when selected these can identify artifact components present in a control condition so that they can be removed from a condition of interest in the sensor data; this de-noised sensor data may then be input to beamformer or minimum-norm inverse methods. A full list of denoising options are listed in Table 1.

*Dual signal subspace projection (DSSP)* One novel advanced denoising method developed in our lab is now available as part of the workbench in the newest releases of NUTMEG. This approach, DSSP (Sekihara et al., 2016) acts by defining a signals in both the space domain as well as the time domain (Sekihara et al., 2016). By generating these two data matrices, DSSP projects the data matrix onto the subspace that is orthogonal to the interference subspace, and removes this interference signal with a constant presence in the data matrix. We have validated this algorithm using both simulated and real data (Cai et al., 2019) and have shown superior artifact rejection in the sensor data through DSSP when benchmarked against other methods, such as signal subspace projection (tSSS). We now provide this option for cleaning the data for both time-series and time-frequency analyzes in the latest release of the workbench.

## Computation of Forward Model

Once the sensor data is preprocessed, the user has the option to generate lead fields from sensors loaded in from the dataset, using a forward model and information from the individual brain structure and MEG channel locations (and therefore generating a gain matrix), through selection of the “Obtain Lead Field” button. NUTMEG provides several options to the user for defining the forward model, and built-in support for computation of sensor lead fields and the gain matrix based on single sphere and multisphere head models. The sphere center can either be specified manually, or loaded from a head model file created from CTF’s localSpheres command line function. The current iteration of NUTMEG permits source localization across the whole brain volume, although calculation of lead fields using more computationally intensive boundary element method (BEM) head models is provided via integration between NUTMEG and either the Helsinki BEM (Stenroos et al., 2007) or the OpenMEEG (Gramfort et al., 2010) toolboxes. NUTMEG includes functions for importing tissue surface meshes from either BrainSuite or BrainVisa MRI segmenting software, thereby presenting the user with a complete BEM pipeline.

For EEG datasets, a multisphere model can be generated using a NUTMEG function that adjusts sphere centers to minimize the difference between the forward potentials generated for a few sparsely sampled points using the multisphere method and those derived using the BEM.

## Inverse Methods: Time-Series (Evoked) Source Analysis

**FIGURE 5:**
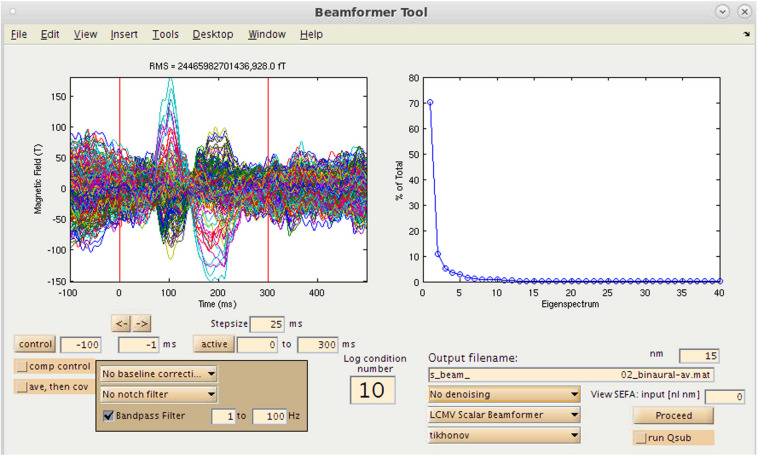
Time-series source estimation GUI. On the left, sensor overlay of the averaged dataset is visible. On the right, parameters for source space reconstruction (in this case, eigenvalues plotted for the eigenspace vector beamformer). Filtering options, time window selection, method of source inversion, other denoising methods, sensor covariance regularization are also selectable.

One option available to the user in NUTMEG for source analysis is the ability to localize evoked magnetic fields (e.g., AEF, SEF, and VEF) generated from averaging of sensor data using a variety of inverse methods. This is selected via the “Source Analysis: Time Series” button in the Main Command window. This brings up the Beamformer Tool GUI (Figure 5) that allows for an interactive and GUI-driven method for users to define their desired baseline period and assign the type of inverse solution to be used.

Many variants of popular inverse methods for source localization of M/EEG data are included in NUTMEG: beamformer, minimum norm, and Bayesian. Furthermore, NUTMEG is stylized to allow easy drop-in and incorporation of newly-developed inverse methods into the associated menu choices. The use of the time-domain LCMV beamformer (Similar to SAM, Vrba and Robinson, 2001) for localizing both the oscillatory power changes over many time-frequency windows as well as evoked responses (ERF/ERPs) is well supported in NUTMEG. Minimum-norm methods that are supported include sLORETA (Pascual-Marqui, 2002) and dSPM (Dale et al., 2000). Several Bayesian methods have been developed by our group to improve source estimation and allow denoising of data, including Champagne (Owen et al., 2012a, b), SAKETINI (Zumer et al., 2007), and NSEFALoc (Zumer et al., 2008). Bayesian methods denoise and localize data in one step, resulting in improved spatial specificity and reduced sensitivity to correlated sources. A full list of the inverse methods available for evoked source analysis can be found in Table 1B and are explained in detail in our previous publications (Dalal et al., 2011).

Covariance Optimization Garnering Noise for Active Cancelation One of the philosophies of NUTMEG is to make a constellation of inverse methods for source solutions available to the user, and in our lab we have developed several such tools (NSEFALoc, SAKETINI, et cetera) that we have readily implemented into the workbench. One major revision to the NUTMEG 4. + release of the software is the inclusion of several novel approaches, including scanning algorithms for source reconstruction. COGNAC (Cai et al., 2018a) has been readily applied and tested to simulated, MEG, and EEG datasets and is now available in the latest release of the software. Here, probabilistic generative modeling is used to describe the sensor data, which partitions source contributions in the sensor data from a given location from contributions to that point in space from neighboring locations, enabling learning of sensor noise without the need for baseline or pre-stimulus data. We find application of COGNAC to several datasets to be superior to more gold-standard means of source localization (beamforming, sLORETA). Given the high utility of this tool in source imaging, we now include it in our latest releases of the NUTMEG workbench.

*Smooth Champagne* One popular beamformer available in NUTMEG is champagne, which uses an empirical Bayesian framework to yield sparse source solutions to the inverse problem (Owen et al., 2012b). Recent developments in our lab have acted to improve the fidelity of this technique, and are now available in the NUTMEG workbench. One of these, which introduces kernel smoothing and hyperparameter tilting into the source solution we refer to as Smooth Champagne (Cai et al., 2018b). We demonstrate that Smooth Champagne is highly robust to noise, interference, and the resolution of highly temporally correlated brain sources for both MEG and EEG. Like COGNAC, this tool is now available in the most up-to-date releases of NUTMEG for users to apply.

Once the particular inverse solution method parameters are assigned by the user, the source analysis is run and a results file (s_beam^∗^.mat) is generated that can be opened by the user in the Visualization Tool interface (see below).

## Inverse Methods: Time Frequency (Induced) Source Analysis

**FIGURE 6:**
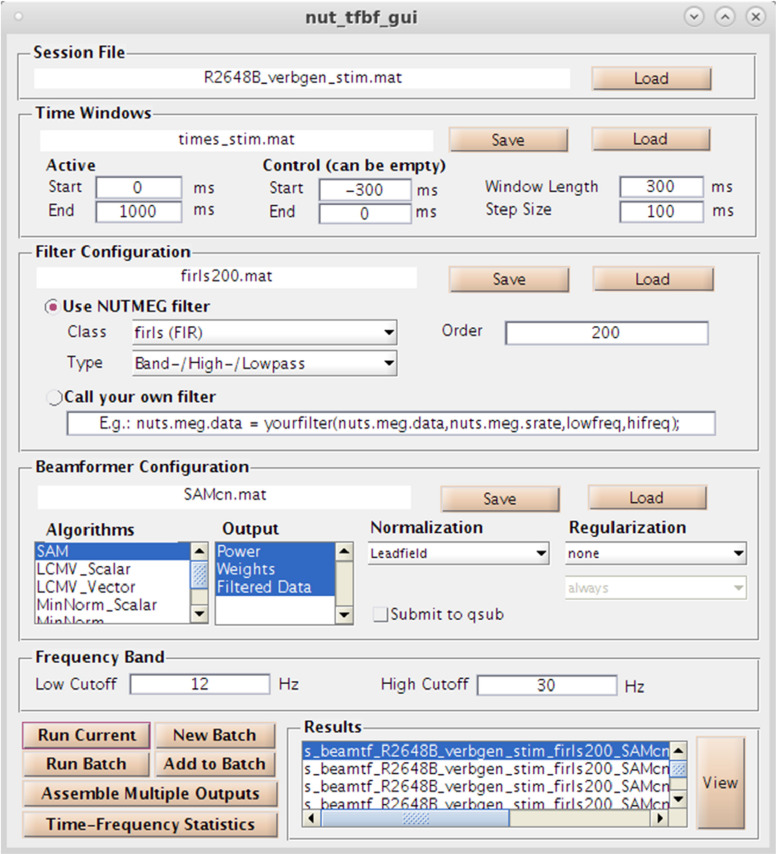
Time-Frequency Beamformer GUI. Specification of time-windows, frequency band, filtering, and source reconstruction algorithms are all available.

As an alternative (or complementary analysis) to evoked activity, NUTMEG provides the option to reconstruct data in the time-frequency domain to evaluate induced (e.g., non-phase locked) changes in oscillatory dynamics using both GUI and command line functions. Selection of the “Source Analysis: Time Frequency” button in the Main Command Window brings up the Time-Frequency Beamformer GUI (Figure 6), an interactive way to define beamformers for source reconstruction in the NUTMEG toolbox. User options for customized time-window definition (length, duration, and overlap), frequency band (e.g., 8–12Hz, 12–30Hz), filtering techniques [e.g., finite-impulse response (FIR), Butterworth], and beamformer method (e.g., SAM, sLORETA) are available, with options to import custom filters/beamformers if the user chooses to do so. The NUTMEG time-frequency pipeline proceeds in three steps (described in more detail in Dalal et al., 2011). First, sensor data is passed through a series of filter banks and partitioned into frequency bands (e.g., alpha 8–12Hz, beta 12–30Hz) and overlapping time windows (e.g., 250 ms windows with 50 ms overlap), pre-defined by the user in the associated GUI inputs or via user-created variables in Matlab files that can be selected within the GUI. From these windows a covariance matrix and source weights are used to estimate power changes in oscillatory activity in each window, which are then finally assembled into a single file (s_beam_timef^∗^.mat) for results visualization. Interrogation of the time-frequency reconstruction by the user can be visualized at the single subject level or as group averages. A full list of the inverse methods currently available for time-frequency optimized source analysis can be found in Table 1C, and each of these processes is explained in more detail below in the visualization and statistics sections, respectively.

## Functional Connectivity Analysis

**FIGURE 7:**
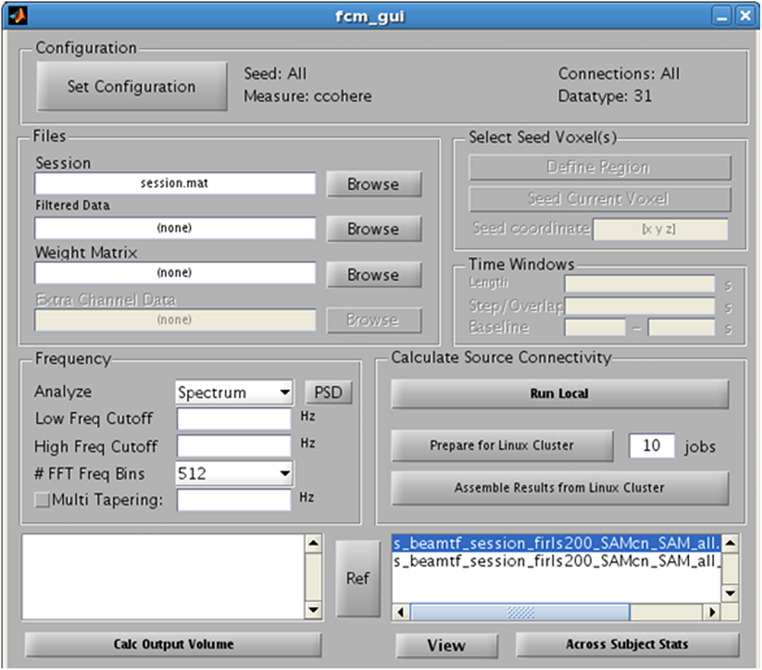
Functional Connectivity GUI. Allows for the selection of the type of connectivity metric, frequency band of interest, and regions of interest.

Finally, as an alternative to examining evoked or induced changes in oscillatory power, NUTMEG offers a functional connectivity map (FCM) workbench that enables the localization of FC among brain areas from EEG and MEG recordings. NUTMEG computes FC by combining source localization algorithms with measures of FC between those sources. First, the user undertakes an estimate of oscillations across networks at each voxel by calculating the linear combination of the sensor data matrix with a spatial weighting matrix obtained with the solutions and steps outlined in 5B. Next, the user can enable, or select, the “FCM” button on the Main Command Window, which brings up the FCM GUI interface (Figure 7).

In the FCM GUI, the user can then set the desired configuration parameters for the FC analysis. The current instantiation of NUTMEG’s FCM tool relies on “bivariate” measures of FC, which requires the user to define both a “seed” and the “target” (or “connection”) regions in the configuration tool. These seeds may be defined voxelwise and then averaged across every target in the grid (what is called “Global Connectivity”; see Guggisberg et al., 2008; Hinkley et al., 2011), or can be selected as a pre-defined Region of Interest (ROI) by manually drawing the VOI on a template brain or using labels from an anatomical atlas (such as the AAL atlas; Tzourio-Mazoyer et al., 2002) or, at an even more coarser level, across an entire cerebral hemisphere.

Functional connectivity estimates can be run on both task-based (e.g., event-locked multi-trial data) and “resting-state” datasets where no significant event occurs (e.g., Dubovik et al., 2012, continuous single-trial data). NUTMEG includes a variety of FC measures out of the box, including imaginary coherence (Nolte et al., 2004), magnitude squared coherence, phase lag index (Stam et al., 2007), amplitude envelope correlations (Brookes et al., 2011), and general lagged coherence (Pascual-Marqui et al., 2011). These algorithms are efficient enough to run on a local workstation, but also may be distributed across a parallel computing grid. Output images can then be visualized in the NUTMEG viewer at a single-subject level, or piped into the NUTMEG statistics interface for group analysis.

## Statistics

**FIGURE 8:**
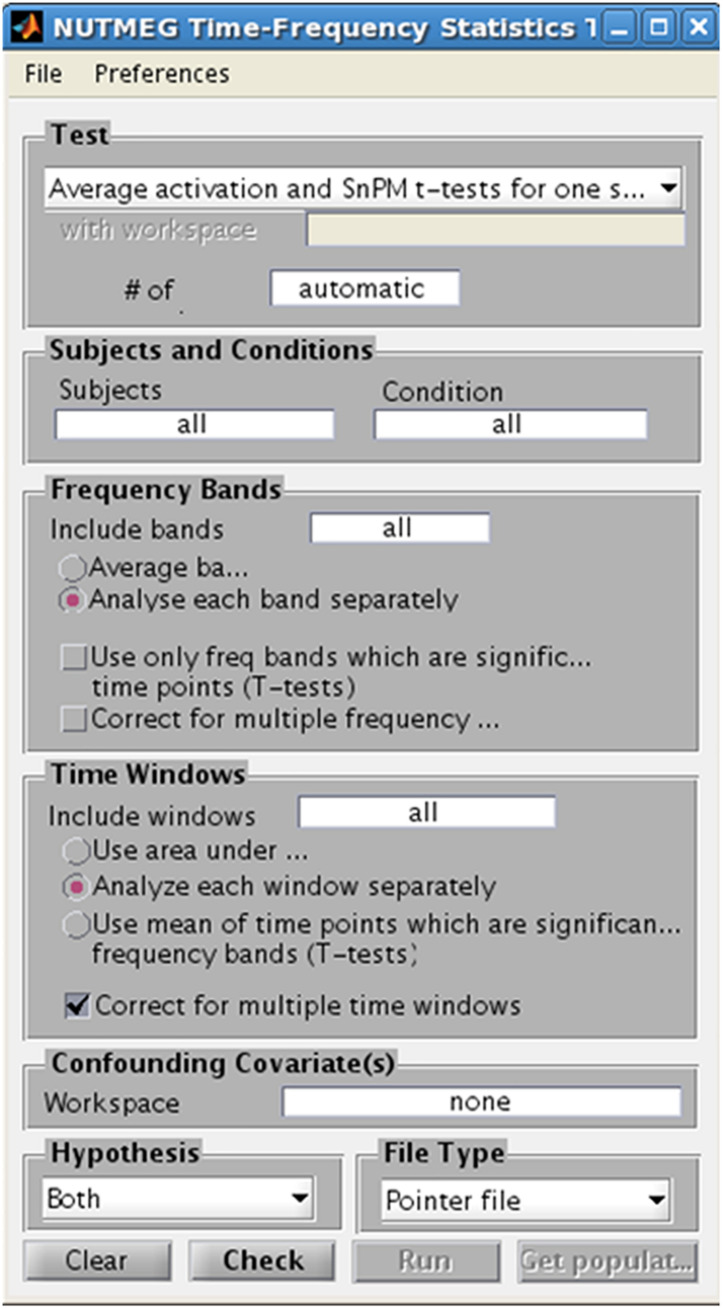
Time-Frequency Statistics Tool, showing various options for calculating statistical significance across subjects for source time-frequency maps.

In studies with considerable sample size (*n* > 5), NUTMEG provides the user with the option to run a variety of voxelwise descriptive and inferential statistics using the Statistics Tool, selected through the Main Command Window (Figure 8). Here, the user selects the normalized s_beam reconstructions, or a “pointer” file that specifies path and filenames of a group of normalized reconstructions generated in a prior step within the visualization tool (see next section), to assess statistical significance across subjects. Once the individual subject files are selected, conditions and groups can be specified and the desired statistical test selected. NUTMEG currently uses statistical non-parametric mapping (SnPM; Singh et al., 2003), which does not depend on an assumption of having normally-distributed data, and is robust for as few as 5 subjects (though having more subjects will allow detection of weaker effects). Current statistical tests available in NUTMEG include grand-mean averaging, one- and two-sample (paired and unpaired) *t*-tests, correlations between power change/FC values with extrinsic (e.g., behavioral) variables, and multi-level ANOVAS. The NUTMEG statistical tool also provides GUI selection options for collapsing across or correcting for significant frequency bands (as in Guggisberg et al., 2010) and time windows.

Since variance estimates can be noisy, variance maps are smoothed with a 3D Gaussian kernel (generally 2 cm). A distribution of pseudo-*t* statistics is created from 2^N permutations of the original N subjects by inverting the polarity of the power change values for some subjects (2^N possible negations) and then finding the current maximum pseudo-*t* value among all voxels and time windows for each frequency band. The significance of each pseudo-*t* value is calculated from its position from a distribution of these maximally permuted pseudo-*t* values. Computed statistical probabilities are formulated as tomographic statistical maps that can be displayed in the NUTMEG visualization tool, and can be thresholded in a variety of different ways to allow for statistical exploration of data. Automatic correction for multiple comparisons across voxels and time-windows is saved in the result file and includes family-wise error rate (FWER), false discovery rate (FDR), and a spatial cluster correction. These thresholds can then be applied to the tomographic statistical map, and changed dynamically using drop-down selections, within the visualization tool to reflect both level of correction for multiple comparisons. Additionally, the desired alpha error rate and related statistical value (e.g., *T*-statistic, *r*-score) can be changed dynamically in the visualization tool for both positive and negative tails of the distribution.

## Visualization of Results

Both visualization of single-subject (FC, power change) and multi-subject (group statistics) data can be viewed using the NUTMEG Results Viewer (Figure 2B) accessible through the Main Command Window. Loading up an s_beam^∗^.mat file will produce a tomographic map overlaid on either the native-space MRI (in the case of single-subject data) or a canonical anatomical brain (for spatially normalized and group data) using the SPM visualization engine. Using this orthogonal-slice navigator, the researcher can explore the source reconstructed dataset or statistical map in 3D space, while an extra, integrated GUI allows the user to explore the dataset over time by displaying the virtual sensor time course for the voxel selected on the SPM navigator. For time-frequency analysis the virtual sensor data plot is replaced by a time-frequency image of the power for the selected voxel. Additionally, the Results Viewer allows the user to spatially normalize a source-space map by taking the transformation matrix from the subject’s T1-wieghted anatomical MRI and apply it to the source-space volume, using SPM normalization functions (Dalal et al., 2008). Normalized source-space map activations can then be displayed on a normalized rendered brain surface. This is performed by selecting the “normalize functional data” in the lower right panel of the timeseries viewer (left side). Once normalized, data from multiple subjects can be loaded into the MATLAB workspace (“File Browser” sub-menu) and their file locations, conditions and group designations can be made and saved to a single MATLAB “pointer” file for subsequent analyzes in the statistics tool.

**FIGURE 9:**
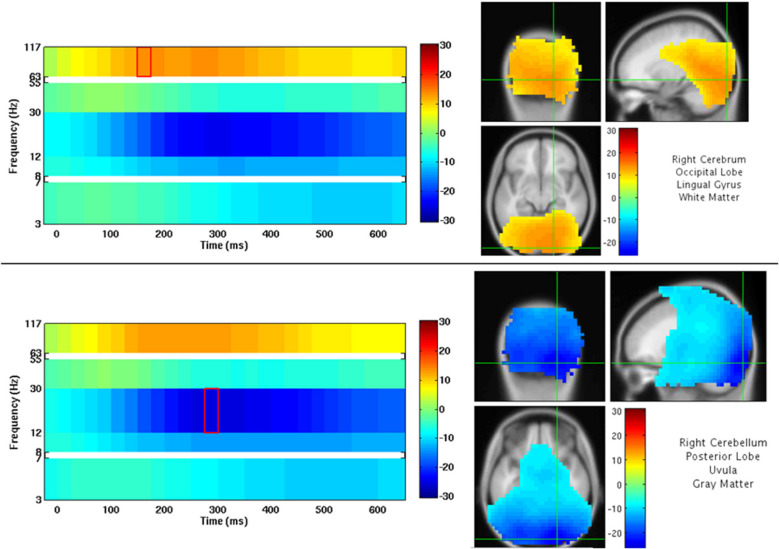
NUTMEG Result Viewer for a time-frequency group analysis (one-sample *t*-test) thresholded at *p* < 0.0005 uncorrected. Time-frequency spectrogram for the voxel highlighted by the crosshairs in the MRI viewer are displayed on the left.

An example of NUTMEG’s visualization of a group analysis (one-sample *t*-test, thresholded at *p* < 0.0005 uncorrected) is shown in Figure 9. Here, a group of subjects viewed faces projected onto a screen. The activation pattern overlay on top of a MRI in the SPM8 Visualization Engine is on the right, and a time-frequency map is presented on the left. The crosshair over the MRI and statistical/tomographic map indicates the voxel in which the time-frequency decomposition is displayed.

Thresholding of the statistical map can be done in a variety of ways in the results viewer. For a single subject, contrast ratios (raw Power for time-series analysis, or a pseudo-*F* ratio contrasting activation and baseline in a time-frequency reconstruction) are selectable from a drop-down window, with the user manually defining the type of threshold to use in a type-in box. These intensity values can be normalized from a scale of 0–1000, and both abscissa and ordinate scales on the time-frequency plots can be adjusted manually to focus on specific portions of the data period. For group (statistical) maps, the drop-down threshold window expands to options where raw scores (*T*-value, *F*-value, et cetera), uncorrected *p*-values or corrected *p*-values (FWE/FDR) can be selected, with the cut-off alpha level adjusted by the user for both positive and negative test values as desired. This aids in exploring the statistical map not over space and time, but also across levels of significance.

Neural activity can also be projected on a 3D brain surface imported from BrainSuite from within the results viewer. There are a number of options to for exporting the data in a format that can be viewed in third-party packages. Export into ANALYZE format which can be then further manipulated in CarTool, mri3dX^3^, DataViewer3D (Gouws et al., 2009), and MRICro (mricro.com) are available. Extensions of the code from the MATLAB to Python language are in place and support additional viewing tools via Xipy^4^.

We will now provide demonstrate these steps in practice on three datasets collected on a 275-channel CTF biomagnetometer.

## NUTMEG in Practice

In closing, we present two tutorials outlining the step-by-step process for: (1) how to generate source reconstruction maps of evoked fields in NUTMEG, and (2) how to reconstruct induced, non-phase locked sources in the time frequency domain in NUTMEG. Specific examples will use the same dataset (described below) and follow the workflow outlined in the beginning of this article.

Datasets used in this tutorial are available for public download in both raw and reconstructed (for group analysis purposes) format at the NUTMEG NITRC website^5^. A dataset consisting of MRI (individual subject T1-weighted MRI) and MEG (single run of face/no-face paradigm, see below) were collected from 39 healthy control subjects. MRI data was collected on a Siemens 3.0T scanner using standard anatomical MRI protocols (Hinkley et al., 2019). MEG data was collected using a 275-channel CTF MEG biomagnetometer. In brief, randomized trials of both face and non-face stimuli were presented foveally on a black background (subtending 12 and 9 degrees of vertical and horizontal visual angle, respectively) requiring the subjects to respond to either “face” or “scrambled face” via button press. 100 neutral face stimuli (Chadick and Gazzaley, 2011) were equated for gender and transformed to gray scale while 100 non-face stimuli were created by randomly shuffling locations of 25 × 25 pixel regions within each face image in MATLAB (200 trials total). A black oval layer masked both face and non-face stimuli to obscure regions around hairline and ears. Stimulus duration (700 to 1100 ms) and inter-stimulus-onset (1.75 s to 2.15 s) were randomized for each trial.

Datasets were pre-processed outside of NUTMEG using CTF software in order to meet the following pre-processing criteria: removal of bad channels and trials with excessive movement (<5 mm in run) or noise (signal > 1.5 pT), 3rd order gradiometer correction, bandpass filtered (3–117 Hz), and create a multiple spheres head model prior to source analysis. Correctly-responded trials were then equated for each stimulus type in a movement- and artifact-free epoched dataset. These steps were performed prior to the source analysis outlined below. For both examples, MATLAB paths were set to contain the recent NUTMEG release and SPM8 toolboxes. We begin both examples following opening the main NUTMEG: Neurodynamic Utility Toolbox for MEG and SPM8 visualization windows (Figure 2A). Specific buttons for the GUI are presented in italicized parentheses for each example.

### NUTMEG in Practice, Example 1: Source Localization of Visual Evoked Fields in a Single Subject Using Champagne

In order to localize visual evoked fields from this dataset, we first average the dataset using CTF tools prior to analysis in NUTMEG. Beginning with Step Two (above) we import the subject-specific native space MRI, normalized space MRI and fiducial markers (via headshape) using the Coregistration Tool (*Coregister MRI*) in Nutmeg. Following data import (*Load MEG/EEG Data*) we then check to make sure the averaged sensor data looks reasonable (*View/Select MEG Channels*) and generation of lead fields from the sensor data loaded in (*Obtain Lead Field*) we then import the dataset’s marker file (*Special→Import Markerfile*) to load and select the triggers for this dataset. The timeseries source analysis tool is then selected from the main GUI (*Source Analysis: Time Series*) where several options for beamforming reconstruction become available. We select the default settings for the Champagne beamformer (Owen et al., 2012a, b) and apply a 1–100 Hz bandpass filter to data. Selecting the Proceed button allows us to generate the image, which is saved out in a s_beam^∗^ file that can be loaded up in the Nutmeg results viewer.

**FIGURE 10:**
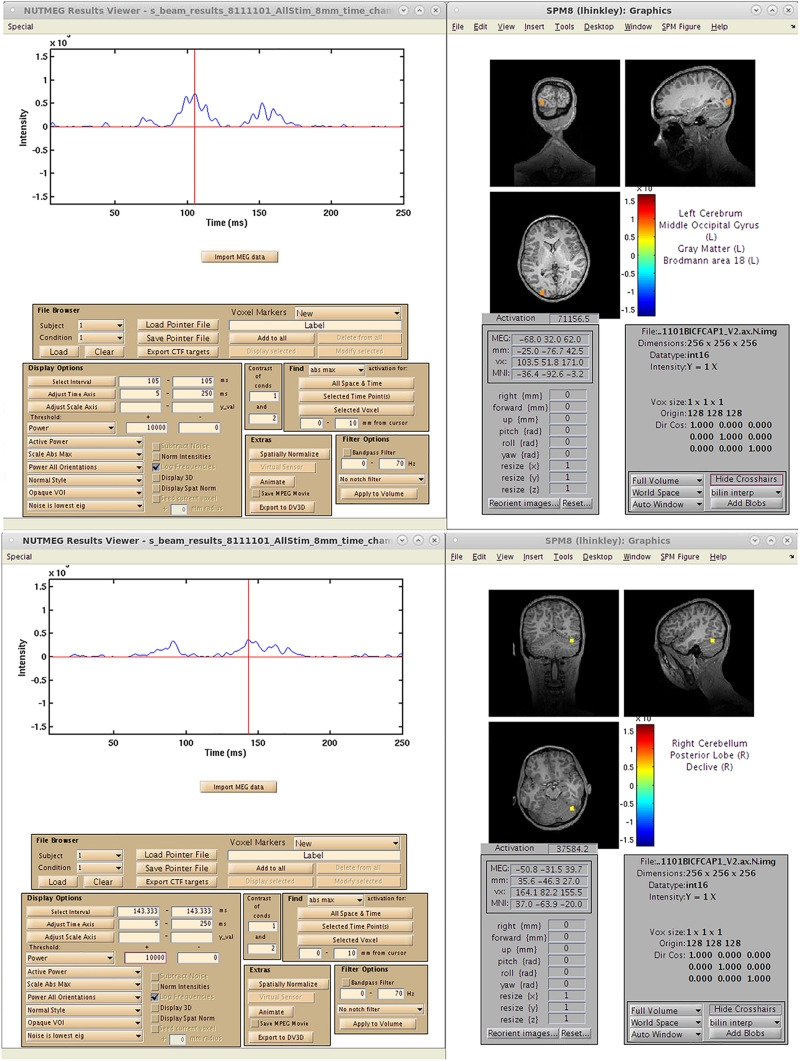
Results from a single-subject reconstruction using the Champagne beamformer in NUTMEG for response to visual stimuli (Nutmeg In-Practice, Example 1). Localized fields for both primary visual cortex (∼92 ms, **A**) and higher-order visual fields (142 ms, **B**) are both shown.

A single-subject VEF is shown in Figure 10. Here, we are able to localize three visual evoked fields following stimulus presentation. The first, at 92 ms post-stimulus, localizes to the lingual gyrus of primary visual cortex (V1) coincident with the visual evoked response (Figure 10A) around 100 ms. The second responses, localizing to the left (151 ms, Figure 10A), and right (147 ms, Figure 10B) middle occipital gyrus (MOG), correspond to a later, M150 response identifiable in higher order visual and extrastriate fields. These results illustrate that the time-series beamformers implemented in NUTMEG, including Champagne, can reliably localize responses in primary sensory cortices.

### NUTMEG in Practice, Example 2: Source Localization of Induced Changes in Visual Cortices Using a Time-Frequency Optimized Beamformer and Group Statistics

Similar steps for data import and preprocessing used in the first example (evoked fields) are used for the reconstruction of induced, non-phase locked sources in the time-frequency domain. Here, instead of using averaged data, single-trial (non-averaged) data is used for the time-frequency optimized beamformer.

We follow Step 1 (as above) to import individual MRI and normalized images (Figure 3) and load the associated MEG dataset, automatically reading fiducial coordinates from the multiple spheres head model located within the MEG file directory. MRI data was normalized using functions within the SPM8 toolbox prior to import. Fiducial locations were visually inspected after re-selecting “Coregister MRI.” Steps 3 and 4 were followed to inspect the sensor data and, as both structural and functional data are available for an individual, create the forward model based on position of the sensor montage relative to MRI landmarks.

As the current study requires comparison of two visually-presented conditions, both trial types are included in the epoched CTF MEG dataset. For this example, we compare our experimental (Face), and control (Non-face) conditions over each time window, localizing brain regions that are specific to face identification and not simple visual processing. Trial types are identified using their stimulus markers via the “Import CTF marker file” drop down option in the “Special” menu (Figure 3B). The individual’s structural and functional data, lead field calculation for each sensor, and the specification of “active” or “control” marker type in the beamforming calculation was then saved (via “save session”) to utilize in source localization and for the convenience of returning to an already-associated dataset if other analyzes are desired in the future. Because the contrast of interest is the differential response to two stimuli, our time windows of interest for active and control are identical — spanning stimulus presentation through end of trial. Had our aim been simple sensory response activation (as in Example 1), active and control stimulus markers would be identical, with active time window through post-stimulus trial duration and the control as a static, pre-stimulus time period. Window lengths and frequency band(s) can be custom-defined in the time-frequency dependent on the sampling rate of the dataset acquired, using the Nyquist limit as a guideline. To initiate the induced time-frequency beamforming analysis, we select the lower “Source Analysis: time-frequency.” option which brings up the time-frequency GUI (Figure 6). Here, we specify our Active (i.e., experimental) and Control window length, time window overlap, beamformer algorithm and frequency band(s) of interest (Figure 6).

In the example study, source localization of 39 participants’ data was batched via NUTMEG command line functions, and run over 5 frequency bands of interest across sliding time windows that covered the trial period, the size of which varied according to bandwidth. Once source localization completed, creating multiple files for each subject, these were consolidated into one result file per individual (“Assemble multiple outputs” selection in the source localization GUI).

After the consolidated result file was created for a single subject, the “View Results” selection within the main NUTMEG menu visualizes the single subject result across the time and frequency bands analyzed, and also provides a mechanism to read in multiple subjects’ localized data into the NUTMEG workspace by indexing the subsequent subject number and corresponding condition and selecting “Load” in the File Browser portion of the viewer. After reading in each of the 39 participants in this section, a pointer file was created and saved that included all subjects’ filenames, paths, subject numbers, and condition information associated with each individual source localization result file. This pointer file is used in the group analysis run via the “Statistics” button on the main NUTMEG window.

**FIGURE 11:**
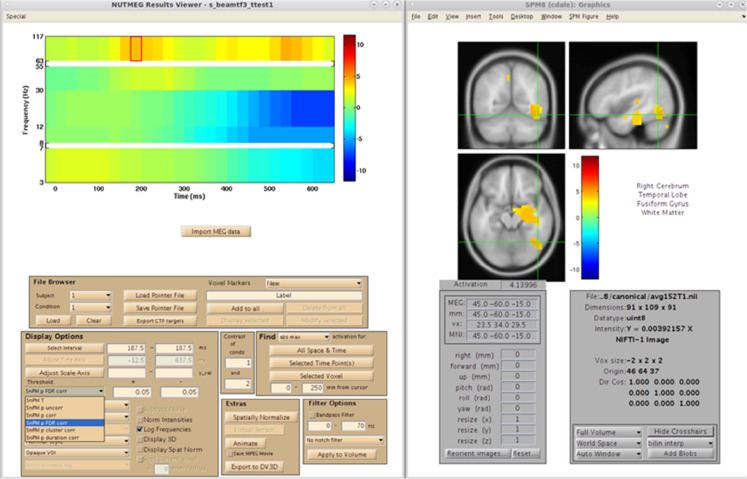
Results from the group analysis using the time-frequency optimized beamformer comparing face to non-face conditions (Nutmeg In-Practice, Example 2). An early increase in high-gamma power (63–117 Hz) localized to the right fusiform is greater in the face condition around 187.5 ms following stimulus presentation.

After reconstruction of all 39 subjects, this pointer file is imported into the NUTMEG Statistical GUI for within-group results of the Face > Non-face contrast. This creates a single file (^∗^ttest1.mat) which can be loaded in the NUTMEG results viewer (Figure 11). The group analysis produces an average activation map for the active (Face) relative to the control (Non-face) condition for the group using a one sample, two-tailed, SnPM *t*-test, with a statistical threshold of *p* < 0.05 under an FDR correction for multiple time windows. This group result reveals significant induced response in high gamma band activity, Faces > Non-faces, in right fusiform gyrus.

## Discussion

The M/EEG community is rapidly shifting and expanding, and as NUTMEG now is well into its fourth version, a large focus of our development is centered on being able to incorporate and integrate advanced functionality to meet these needs. A prime example of this is making sure that NUTMEG integrates and interfaces with many of the other software packages available to analyze M/EEG data, allowing the user to seamlessly move datasets and analyzes between the platforms to benefit the most from the strengths of each. With resurgence in both EEG and ECOG research for the purpose of performing source analysis, much work is being done to integrate these types of data into the analysis framework of NUTMEG. While pipelines exist to import more sophisticated head models (like BEM) for source localization in NUTMEG, we are currently working on options for the user to apply these methods directly in the workbench, as well as options for both volume and surface-based source reconstructions. Our own lab and others continually refine and improve inverse methods for the purposes of improving source localization, and our developers are continually at work to include these types of novel techniques available to the NUTMEG user. In the same vein, statistical metrics (including corrections for multiple comparisons at the voxelwise level) and functional connectivity methods continue to evolve and will be added in future versions of the software. Expanded options for multimodal data integration (for example, voxel-based morphometry, and diffusion tensor imaging) will be available in future versions of the software. We also plan to expand the options available for the user at early stages of data preparation, including trial selection for both artifact rejection and trial-by-trial analyzes. Furthermore, the integration of multiple sensor types (e.g., magnetometers and planar gradiometers) in a way that would add value to the robustness of the source solution is another area of robust research in MEG (see Gramfort et al., 2013; Engemann and Gramfort, 2015), and could potentially be integrated into next-generation releases versions of NUTMEG.

Many of the programming environments used for the analysis of neuroimaging data (including NUTMEG) are proprietary extensions of existing computing environments (in our case, MATLAB) optimal for applied mathematics and not imaging analysis *per se*. While MATLAB is the most popular computing platform in neuroscience, it is becoming increasingly clear that newer programming environments may additionally serve data analytics. In order to expand NUTMEG into a true open-source environment, there is a need for future generations of the workbench to be coded in programming languages that are more accessible. Python^6^ is a logical choice for next-generation software development in neuroimaging, as it is high-level, object-oriented and interactive. As software development in Python has proven fruitful in other M/EEG analysis software packages (most notably MNE-Python). we plan to produce versions of NUTMEG in this programming language, further providing access to the software.

On a final note, MEG is entering a modern “renaissance” at the hardware level. Not only are new biomagnetometer manufacturers (such as RICOH of Japan) entering the scene, but exciting developments in so-called “helium-free” or “room-temp” magnetometers (including the HyQUID system offered by York Instruments and the optically-pumped magnetometers outlined in Boto et al., 2017) provide new potential avenues of data integration for NUTMEG. While this may introduce a unique set of challenges for the regular imaging scientist, as part of our mission, we will ensure that NUTMEG is accessible for every MEG user, regardless of hardware. We welcome and encourage collaborators and developers who wish to contribute to this endeavor.

## Data Availability Statement

The datasets generated for this study are available on the NITRC website (nitrc.org/projects/nutmeg/) or by request to the corresponding author.

## Ethics Statement

The studies involving human participants were reviewed and approved by UCSF Committee on Human Research. The patients/participants provided their written informed consent to participate in this study.

## Author Contributions

LH, CD, and SN contributed to writing this manuscript. JZ, SD, CC, KS, and SN designed the analyses used in the manuscript. AF assisted in collection of the data presented in the manuscript. All authors contributed to the article and approved the submitted version.

## Conflict of Interest

The authors declare that the research was conducted in the absence of any commercial or financial relationships that could be construed as a potential conflict of interest.

## Footnotes

1 https://www.nitrc.org/projects/nutmeg

2 https://www.fil.ion.ucl.ac.uk/spm/software/spm8

3 www.cubric.cf.ac.uk/Documentation/mri3dX

4 https://github.com/miketrumpis/xipy

5 https://www.nitrc.org/projects/nutmeg

6 www.python.org
